# Neuropsychological profiles in children and young adults with spina bifida

**DOI:** 10.1007/s00381-021-05089-9

**Published:** 2021-03-11

**Authors:** C. Rendeli, E. Ausili, R. Moroni, M. Capriati, L. Massimi, C. Zanetti

**Affiliations:** 1 Dipartimento Scienze della salute della donna, del bambino e di sanità pubblica – Unità Operativa Spina Bifida e Uropatie Malformative, Fondazione Policlinico Universitario A. Gemelli IRCCS, Rome, Italy; 2 Direzione Scientifica – Fondazione Policlinico Universitario A. Gemelli IRCCS, Rome, Italy; 3 Scienze dell’invecchiamento, neurologiche, ortopediche e della testa-collo, Rome, Italy

**Keywords:** Spina bifida, Myelomeningocele, Hydrocephalus, General Intelligence QI, Neuropsychological profiles, Executive function assessment

## Abstract

**Purpose:**

A total of 43 Italian children, aged between 6 and 16 years, diagnosed with spina bifida, myelomeningocele, and shunted hydrocephalus have been described clinically and completed a neuropsychological battery in order to evaluate their cognitive, personality, and behavior profile.

**Methods:**

Enrolled children underwent cognitive assessment by means of the Weschler WISC-IV cognitive test and assessment of the attention sustained through the LEITER test. In addition, parents were asked, in order to obtain a personality and behavior profile of the children, to fill in a “CBCL 6-18 years” questionnaire and to fill in a Barthel Index questionnaire.

**Results:**

Processing Speed Index of the WISC-IV QI scale was statistically significant (*p* = 0.027), with the highest value presented by autonomous patients (95.8 ± 12.8) and the lowest by patients using a wheelchair (75.5 ± 19). WISC-IV QI mean value is 98 (±15.7) for lipoma patients and 78.7 (±17.6) for LMMC and MMC patients (*p* = 0.001). In more detail, Perceptual Reasoning (*p* < 0.0005), Working Memory (*p* = 0.01), and Processing Speed Index (*p* = 0.001) highlighted a significant difference between the groups. The attention sustained subscale of the LEITER presented a mean of 6.9 (±3.1) for lipoma patients and a men value of 4.6 (±3.1) for LMMC and MMC patients (*p* = 0.024). Patients with hydrocephalus had statistically significant worse cognition and autonomy (Barthel Index) score (*p* < 0.001) compared with those without hydrocephalus, and normal scores regarding attention and depression scales.

**Conclusion:**

These results can be useful in planning dedicated therapeutic protocols such as suitable rehabilitation treatments, speech therapy, psychomotor skills, and cognitive enhancement and to develop prevention protocols particularly tailored for children with hydrocephalus who appear to have the more deficient skills.

## Introduction

Spina bifida (myelomeningocele) is one of the most complex birth defects compatible with life, with an estimated prevalence between 3.06 and 3.13 cases per 10,000 live births [1].

Myelomeningocele is the most common form of “spina bifida,” but other forms of open and closed lesions exist, including lipomyelomeningocele, diastematomyelia, diplomyelia, myelocystocele, myeloschisis, and fatty filum [2]. Spina bifida is the most common central nervous system birth defect encountered by the pediatric neurosurgeon. It is defined by characteristic development abnormalities of the vertebrae and spinal cord and associated changes in the cerebrum, brainstem, and peripheral nerves. As a result of denervation, muscle imbalance ensues and can result in abnormalities at the hip, knee, and foot. Anesthesia of various portions of the skin can lead to pressure sores, particularly later in life. Anorectal neuropathy may cause a variety of defecatory dysfunctions. Urologic abnormalities are also common. These multisystem abnormalities associated with spina bifida contribute to its widely accepted identity as the most complex development defect compatible with long life [3]. With advances in medical care, more individuals with spina bifida are surviving to adulthood [4, 5]. A total of 43 Italian children, aged between 6 and 16 years, diagnosed with spina bifida, myelomeningocele, and shunted hydrocephalus, followed in Spina Bifida Centre of Policlinico Universitario A. Gemelli – Roma, have been described clinically and completed a neuropsychological battery in order to evaluate their profile.

## Materials and methods

A total of 43 Italian children and young adults diagnosed with spina bifida completed a neuropsychological assessment battery in order to have their profiles evaluated. Enrolled children underwent cognitive assessment by means of the Weschler WISC-IV cognitive test and assessment of the attention sustained through the LEITER test. In addition, parents were asked, in order to obtain a personality and behavior profile of the children, to fill in a “CBCL 6-18 years” questionnaire and to fill in a Barthel Index questionnaire. Informed consent was signed by the parents. The protocol for this study was approved by the Ethics Committee of the Fondazione Policlinico A. Gemelli IRCCS, ID number: 3218 (2020).

### Neuropsychological tests

The *Wechsler Intelligence Scale for Children-IV* (*WISC-IV*) is the clinical tool par excellence administered individually, to assess the cognitive abilities of children aged between 6 years and 0 months and 16 years and 11 months. The structure of the scale has been updated to reflect the theory and practice of assessing children, which implies increasing attention to working memory and processing speed. With the WISC-IV, 5 composite scores can be calculated: a total intellectual quotient to represent the complex cognitive abilities of the child, and 4 additional scores: the Verbal Comprehension Index, the Perceptual Reasoning Index, the Working Memory Index, and the Processing Speed Index. The *Test Leiter-R International Performance Scale – Revised* “attention sustained” is defined as the ability to maintain attention for a long time on “boring” tasks. The *Child Behavior Checklist* (*CBCL, Achenbach, 6-18 years*) version for parents is structured around 8 syndromic scales: anxiety/depression, withdrawal/depression, somatic complaints, social problems, thinking problems, attention problems, transgression behavior rules, and aggressive behavior, which are grouped into two other general dimensions, internalization and outsourcing.

### Statistical analysis

The sample has been described in its clinical and demographic characteristics using descriptive statistics techniques. Qualitative variables have been presented with absolute frequencies and percentages (*n*, %), and quantitative variables have been summarized with mean (±standard deviation). Normality of data has been verified using Kolmogorov-Smirnov test. Patients have been classified according to gait (autonomous, wheelchair, and tutor) and according to a dichotomized version of the diagnosis: lipoma, lipomyelomeningocele, and myelomeningocele (LMMC and MMC). Comparisons of categorical variables have been performed with Chi-square test (or Fisher’s exact test), and comparisons of continuous variables have been performed with Mann-Whitney *U* test and Kruskal-Wallis test. A *p* value <0.05 has been considered statistically significant.

## Results

A total of 43 patients, 22 females (51.2%) and 21 males (48.8%), have been enrolled in the study. Mean age of the whole sample was 10.72 years (±3.1) with a minimum age of 6 years and a maximum age of 16. As shown in Table 1, the majority of our patients completed primary school (21, 48.8%) and received a lipoma diagnosis (22, 51.2%) or a myelomeningocele (MMC) (20, 46.5%) or a lipomyelomeningocele (LMMC) diagnosis (1, 2.32%). Hydrocephalus is present in 16 patients (37.2%) and all of them have been diagnosed as myelomeningocele. Ventriculoperitoneal shunt is present in 14 patients (32.6%). Most of the patients were in a condition of autonomous gait (26, 60.5%). Barthel Index mean value was 14.91 (±5.1). Table 2 shows that WISC mean value was 88.83 (±19.1) and, in more detail, the Verbal Comprehension Index mean value was 96.58 (±16.7), the Working Memory mean value was 83.56 (±18.6), and the Processing Speed Index mean value was 90.93 (±17.6). The distributions of categorical variables by gait highlighted a statistically significant difference in Barthel Index (*p* = 0.001) where patients with wheelchair presented the lowest Barthel Index mean value (8.3±4.6) and the highest was presented by autonomous patients (17.1 ± 3.1). Also, the Processing Speed Index of the WISC-IV QI scale was statistically significant (*p* = 0.027), with the highest value presented by autonomous patients (95.8 ± 12.8) and the lowest by patients using a wheelchair (75.5 ± 19). See Table 3 for details. Table 4 presents the comparisons of patients according to diagnosis. WISC-IV QI mean value is 98 (±15.7) for lipoma patients and 78.7 (±17.6) for LMMC and MMC patients (*p* = 0.001). In more detail, Perceptual Reasoning (*p* < 0.0005), Working Memory (*p* = 0.01), and Processing Speed index (*p* = 0.001) highlighted a significant difference between the groups. The attention sustained subscale of the LEITER presented a mean of 6.9 (±3.1) for lipoma patients and a men value of 4.6 (±3.1) for LMMC and MMC patients (*p* = 0.024). Table 5 reports a comparison between patients with and without hydrocephalus about cognition and autonomy. As shown, patients with hydrocephalus had worse cognition score regarding IQ (*p* < 0.001) and autonomy (Barthel Index) (*p* < 0.001).

**Table 1:** Clinical and demographic characteristics of the whole sample (*n* = 43)

	Sample	Percentage
Gender		
F	22	51.2
M	21	48.8
Education		
Primary	21	48.8
Lower secondary	14	32.6
Upper secondary	8	18.6
Diagnosis		
LIPOMA	22	51.2
MMC+LMMC	21	48.8
Hydrocephalus		
No	27	62.8
Yes	16	37.2
VP shunt		
No	29	67.4
Yes	14	32.6
Gait		
Autonomous	26	60.5
Tutor	9	20.9
Wheelchair	8	18.6
	Mean	Std. dev.
Age	10.72	3.065
Barthel Index	14.91	5.079

**Table 2:** Clinical characteristics of the whole sample (*n* = 43)

	Mean	Std. dev.
WISC-IV QI	88.83	19.088
Verbal Comprehension Index	96.58	16.783
Perceptual Reasoning	88.37	20.121
Working Memory	83.56	18.593
Processing Speed Index	90.93	17.667
CBCL		
Internalizing problems	58.65	8.144
Externalizing problems	49.81	7.346
Total problems	50.23	8.106
LEITER		
Attention sustained	5.83	3.263

**Table 3:** Distributions of some continuous variables by gait

Gait	Autonomous			Wheelchair			Tutor			*p*-value
	*n*	Mean	Std. dev.	*n*	Mean	Std. dev.	*n*	Mean	Std. dev.	
Age	26	10.4	3.0	8	12.8	2.6	9	9.8	3.2	0.087
Barthel Index autonomia	26	17.1	3.1	8	8.3	4.6	9	14.6	5.1	*0.001*
WISC-IV QI	26	92.2	14.1	7	75.9	22.9	9	89.3	25.8	0.251
Verbal Comprehension Index	26	98.5	11.9	8	89.0	22.7	9	97.7	22.6	0.719
Perceptual Reasoning	26	93.0	15.5	8	70.6	25.1	9	90.8	20.8	0.054
Working Memory	26	87.4	15.7	8	73.0	17.1	9	81.8	25.0	0.176
Processing Speed Index	26	95.8	12.8	8	75.5	19.0	9	90.6	22.3	*0.027*
CBCL										
Internalizing problems	26	58.8	8.6	8	57.4	6.0	9	59.2	9.1	0.782
Externalizing problems	26	50.2	8.4	8	49.3	5.5	9	49.3	5.9	0.948
Total problems	26	50.3	9.0	8	48.9	4.8	9	51.3	8.2	0.865
LEITER										
Attention sustained	26	6.6	3.0	7	3.4	3.7	8	5.5	3.0	0.079

**Table 4:** Distributions of some continuous variables by diagnosis

	Lipoma			LMMC and MMC			*p*-value
	*n*	Mean	Std. dev.	*n*	Mean	Std. dev.	
Age	22.0	10.4	2.8	21.0	11.1	3.4	0.501
Barthel Index autonomia	22.0	16.4	4.0	21.0	13.3	5.7	0.072
WISC-IV QI	22.0	98.0	15.7	20.0	78.7	17.6	*0.001*
Verbal Comprehension Index	22.0	102.5	14.0	21.0	90.4	17.6	0.063
Perceptual Reasoning	22.0	99.2	16.9	21.0	77.0	16.9	*<0.0005*
Working Memory	22.0	90.7	16.0	21.0	76.0	18.5	*0.010*
Processing Speed Index	22.0	99.8	13.3	21.0	81.6	17.1	*0.001*
CBCL							
Internalizing problems	22.0	58.7	8.8	21.0	58.6	7.6	0.903
Externalizing problems	22.0	50.5	8.5	21.0	49.0	6.1	0.557
Total problems	22.0	50.6	8.8	21.0	49.9	7.5	0.826
LEITER attention sustained	22.0	6.9	3.1	19.0	4.6	3.1	*0.024*

**Table 5:** Distributions of some continuous variables by presence of hydrocephalus

	Without hydrocephalus			With hydrocephalus			*p*-value
	*n*	Mean	Std. dev.	*n*	Mean	Std. dev.	
Age	26	10.3	2.8	16	11.7	3.3	0.167
Barthel Index autonomia	26	16.6	3.7	16	12.0	5.9	*0.010*
WISC-IV QI	26	96.7	15.2	15	75.7	18.7	*0.001*
Verbal Comprehension Index	26	102.8	13.3	16	86.6	17.9	*0.008*
Perceptual Reasoning	26	96.8	16.6	16	75.1	18.9	*0.001*
Working Memory	26	90.7	15.4	16	72.5	18.7	*0.003*
Processing Speed Index	26	97.7	13.6	16	80.2	19.1	*0.003*
CBCL							
Internalizing problems	26	58.0	9.0	16	59.6	7.1	0.622
Externalizing problems	26	50.0	7.9	16	49.0	6.3	0.845
Total problems	26	50.1	8.5	16	50.3	7.9	0.917
LEITER attention sustained	26	6.6	3.1	14	4.2	3.2	*0.023*

Bivariate analyses have shown that within those who are autonomous, there are no significant differences between lipoma and MMC patients in terms of neuropsychological test results, similarly among wheelchair users and among tutor users.

There is no statistically significant difference, for neuropsychological tests result, comparing patients without hydrocephalus and with different gait abilities. However, within patients with hydrocephalus, some tests gave significantly different results between gait ability groups: The Barthel Index shows significantly different results between “autonomous vs. wheelchair” (*p* = 0.035) and “tutor vs. wheelchair” (*p* = 0.017). Perceptual reasoning presented significantly different results between “autonomous vs. wheelchair” (*p* = 0.042) and “tutor vs. wheelchair” (*p* = 0.013). Processing speed was significantly different between “autonomous vs. wheelchair” (*p* = 0.033) and “tutor vs. wheelchair” (*p* = 0.031). LEITER attention sustained presented significantly different results between “autonomous vs. wheelchair” (*p* = 0.024) and “tutor vs. wheelchair” (*p* = 0.049). See Table 6 for details.

**Table 6:** Distributions of neuropsychological results by gait abilities in the hydrocephalus group

	With hydrocephalus									
	Autonomous			Wheelchair			Tutor			*p*-value
	*n*	Mean	Std. dev.	*n*	Mean	Std. dev.	*n*	Mean	Std. dev.	
Age	3	13.7	2.1	6	12.5	3.0	7	10.1	3.6	0.3
Barthel Index autonomia	3	15.7	2.1	6	7.0	3.5	7	14.7	5.9	*0.0*
WISC-IV QI	3	79.7	8.5	5	64.4	14.6	7	82.1	22.2	0.2
Verbal Comprehension Index	3	86.7	8.3	6	81.7	21.5	7	90.7	18.6	0.6
Perceptual Reasoning	3	84.0	9.5	6	58.3	12.1	7	85.7	17.0	*0.0*
Working Memory	3	75.0	11.4	6	68.5	16.4	7	74.9	24.1	0.8
Processing Speed Index	3	92.0	9.2	6	66.3	10.0	7	87.0	22.1	*0.0*
CBCL										
Internalizing problems	3	59.7	11.0	6	57.7	6.9	7	61.1	6.3	0.6
Externalizing problems	3	44.3	9.6	6	50.0	4.5	7	50.1	6.2	0.6
Total problems	3	47.0	14.4	6	48.7	4.9	7	53.1	7.1	0.6
LEITER attention sustained	3	7.0	3.5	5	1.6	1.3	6	5.0	2.8	*0.0*

## Discussion

Spina bifida malformation (SBM) is a neural tube defect originating in the first 30 days of gestation and associated with failure of the caudal end of the neural tube to close. It is a heterogeneous congenital disorder with complex physical and neuropsychological symptomatology. It is associated with developmental anomalies of both the spine and the central nervous system. Different forms of spina bifida can be distinguished from mild to severe. The most common form is myelomeningocele (MMC). Most patients with myelomeningocele present associated hydrocephalus. Hydrocephalus in SBM results from the Chiari II cerebellum and hindbrain malformations, which obstructs the flow of the cerebrospinal fluid (CSF) at the level of the third and fourth ventricles. Surgical management with ventriculoperitoneal shunt implantation (VPS) is a widely used, effective treatment for hydrocephalus. However, consistent follow-up is necessary after VPS, taking into consideration several shunt-related complications including obstruction, fracture, and infection. Also, the endoscopic third ventriculostomy (ETV) procedure is well recognized as an effective alternative to cerebrospinal fluid (CSF) shunt placement and has been the ideal treatment of choice for obstructive hydrocephalus in recent years. As for the neurocognitive aspects, children with hydrocephalus and SBM are more impaired in spatial, math, memory, and concept formation domains, compared to their relative strengths in vocabulary and word reading domains [6, 7]. Within content domains, cognitive strengths involve the learned association and categorization of stimulus information, such as word recognition, vocabulary, and priming abilities. Areas of weakness involve abstract assembly and construction of information, such as coordinating visual perception, mathematical computation, reading and language comprehension, and concept formation tasks [6–8]. The purpose of this study was to observe the psychological and neuropsychological condition of children affected by spina bifida, a condition with a great severity and clinical variety. The study showed that lipoma patients have a more adequate cognitive function development than MMC patients. Moreover, patients with autonomous ambulation (most of them are lipoma patients) presented a normal cognitive development unlike patients in need of a wheelchair or a tutor. Patients that need a wheelchair are the most affected both clinically and cognitively. Considering the components of cognitive processes (WISC-IV), such as Perceptual Reasoning, Working Memory, and Processing Speed Index, the study proved that these skills are preserved in lipoma patients rather than in MMC patients who have difficulties in these skills due to the severity of their pathology. Particularly, the Processing Speed ability is linked with concentration and attention, cognitive flexibility, visual ability, and organizing an autonomous task fast. These results can be useful in planning dedicated therapeutic protocols such as suitable rehabilitation treatments, speech therapy, psychomotor skills, and cognitive enhancement. Cognitive ability scores, such as Working Memory (WISC-IV) and Sustained Attention (LEITER), are in both lipoma and LMMC and MMC patients below normal values and/or slightly below normal values [9, 10].

From a behavioral point of view, the analysis of the questionnaires filled by the parents highlights that no clinical or pathological scores are present in the three scales investigated by the CBCL tool. Results highlight that children, even if they do not have clinical scores, tend to use internalizing defense mechanisms, showing an attitude characterized by greater emotional closure and social withdrawal rather than externalizing attitude (outbursts or aggression).

Study’s results highlighted that children with spina bifida, checked from birth constantly each 6-12 months, have a normal cognitive functioning characterized by difficulties in working memory, which is slightly lower than the normal value, but with a strength in verbal understanding. These findings can guide a better assessment of patients’ needs in order to develop prevention protocols (suitable rehabilitation treatments such as speech therapy, psychomotor skills, cognitive enhancement) particularly tailored for children with hydrocephalus who appear to have the more deficient skills. About the presence of hydrocephalus, the authors observed that these patients have worse score both for cognitive intelligent quotient analyzed with the WISCH scale and for autonomy with the Barthel Index. These results can be explained because patients with hydrocephalus have severe clinical manifestations of spina bifida and also because hydrocephalus causes a worse impact on quality of life and cognitive performances.

## Conclusions

This study underlined that spina bifida patients should also be evaluated for their neuropsychological deficits in order to organize specific rehabilitation programs.

### Availability of data and material (data transparency)

The datasets used and/or analyzed during the current study are available from the corresponding author on reasonable request.
